# Neurophysiological gradient in the Parkinsonian subthalamic nucleus as a marker for motor symptoms and apathy

**DOI:** 10.1038/s41531-024-00848-2

**Published:** 2025-01-03

**Authors:** Elena Bernasconi, Deborah Amstutz, Alberto Averna, Petra Fischer, Mario Sousa, Ines Debove, Katrin Petermann, Laura Alva, Andreia D. Magalhães, M. Lenard Lachenmayer, Thuy-Anh K. Nguyen, Michael Schuepbach, Andreas Nowacki, Claudio Pollo, Paul Krack, Gerd Tinkhauser

**Affiliations:** 1 Department of Neurology, Bern University Hospital and University of Bern, Bern, Switzerland; 2 Graduate School of Cellular and Biomedical Sciences (GCB), University of Bern, Bern, Switzerland; 3 Department of Biomedical Research, University of Bern, Bern, Switzerland; 4 Graduate School for Health Sciences, University of Bern, Bern, Switzerland; 5 School of Physiology, Pharmacology & Neuroscience, University of Bristol, University Walk, BS8 1TD Bristol, UK; 6 Department of Neurosurgery, Bern University Hospital and University of Bern, Bern, Switzerland; 7 ARTORG Center for Biomedical Engineering Research, University of Bern, Bern, Switzerland; 8 Institute of Neurology, Konolfingen, Switzerland

**Keywords:** Parkinson's disease, Parkinson's disease, Predictive markers, Neurophysiology, Neurodegeneration

## Abstract

Sensing-based deep brain stimulation should optimally consider both the motor and neuropsychiatric domain to maximize quality of life of Parkinson’s disease (PD) patients. Here we characterize the neurophysiological properties of the subthalamic nucleus (STN) in 69 PD patients using a newly established neurophysiological gradient metric and contextualize it with motor symptoms and apathy. We could evidence a STN power gradient that holds most of the spectral information between 5 and 30 Hz spanning along the dorsal-ventral axis. It shows elevated power in the sub-beta range (8-12 Hz) toward the ventral STN, and elevated dorsal beta power (16–24 Hz) indicative for the hemispheres contralateral to the more affected hemi-body side. The rigidity response to DBS was highest dorsally on the axis. Importantly, apathetic symptoms can be related to reduced ventral alpha power. In conclusion, the STN spectral gradient may inform about the motor and neuropsychiatric domain, supporting integrative closed-loop strategies.

## Introduction

Parkinson’s disease (PD) is characterized by multi-facetted symptoms affecting the motor and neuropsychiatric, motivational domain^1^. Apathy, describing a pathological reduction in goal-oriented behavior, is highly prevalent in PD and causes major impact on the patient’s quality of life^2,3^. The integrity of subcortical structures linking the prefrontal cortex with the limbic system is critical for orchestrating the motivational drive but can be disrupted by the neurodegenerative process. The behavioural continuum of motivation ranges from apathy on the hypodopaminergic side to impulsive behavior on the opposite end of the spectrum^4,5^. This is conceptually highly relevant for the management of PD patients who undergo deep brain stimulation (DBS) surgery, as the success of this therapy is closely linked to the ability of the clinician to finely titrate both medication and stimulation^6,7^. The subthalamic nucleus (STN) is a principal DBS target in PD and is characterized by a functional subdivision into motor, associative and limbic regions^8–11^. This nucleus further has a heterogenous spectral architecture, suggesting that symptom biomarkers for different clinical domains could be spectrally and spatially differentiated^12,13^. To date, beta activity (13–30 Hz) is the best described neurophysiological motor symptom biomarker in PD supported by a large body of literature^14–26^. In comparison, the role of basal ganglia signals in the context of the apathy-impulsivity spectrum is understudied. Only few data are pointing to an increase in ventromedial sub-beta oscillations (5–12 Hz) and an association of it to trait impulsivity and impulse control behavior, while no studies so far describe a basal ganglia neurophysiological correlate of apathy^27–32^.

Next generation sensing-based DBS is a promising tool to optimally balance both stimulation and dopaminergic treatments, given its capacity of passively monitoring patient’s clinical state via basal ganglia activity recordings^33,34^. For these novel treatment strategies to be as comprehensive as possible, both the spatial and spectral properties of the STN spanning the motor and neuropsychiatric domain need to be understood and integrated in the future workflows^35–37^. To contribute to these developments, the present work systematically investigates the cross regional clinical-neurophysiology of the STN in the context of the motor symptoms and apathy on a large and representative dataset of PD patients using implanted multi-contact DBS leads.

## Results

### Spectral transitions within the STN

**Fig. 1:**
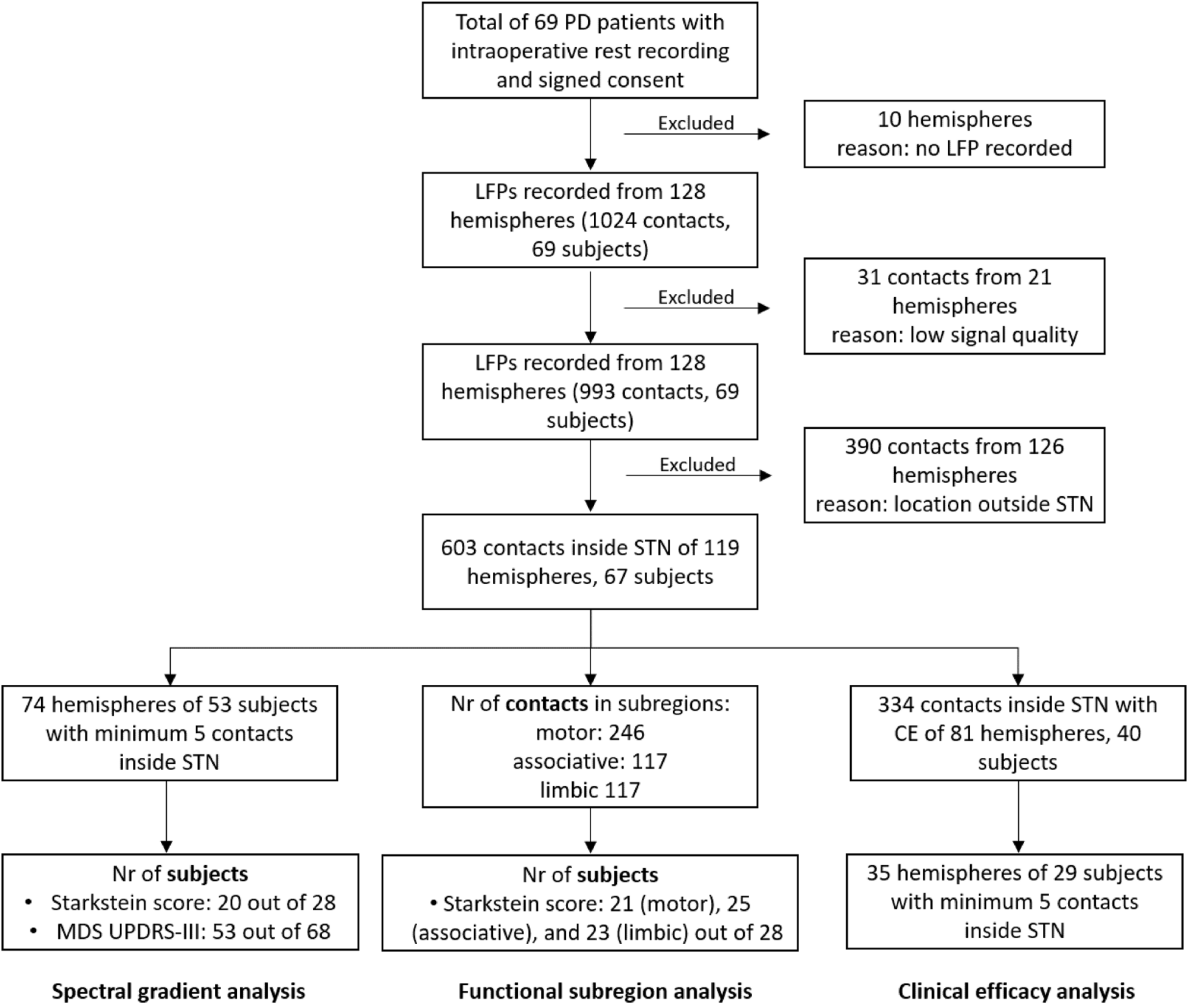
Inclusion and exclusion criteria for the analysis pipelines. Flow chart summarizing the total cohort with inclusion/exclusion criteria of DBS contacts, hemispheres and subjects. PD Parkinson’s disease, LFP local field potential, STN subthalamic nucleus, CE clinical efficacy.

**Fig. 2:**
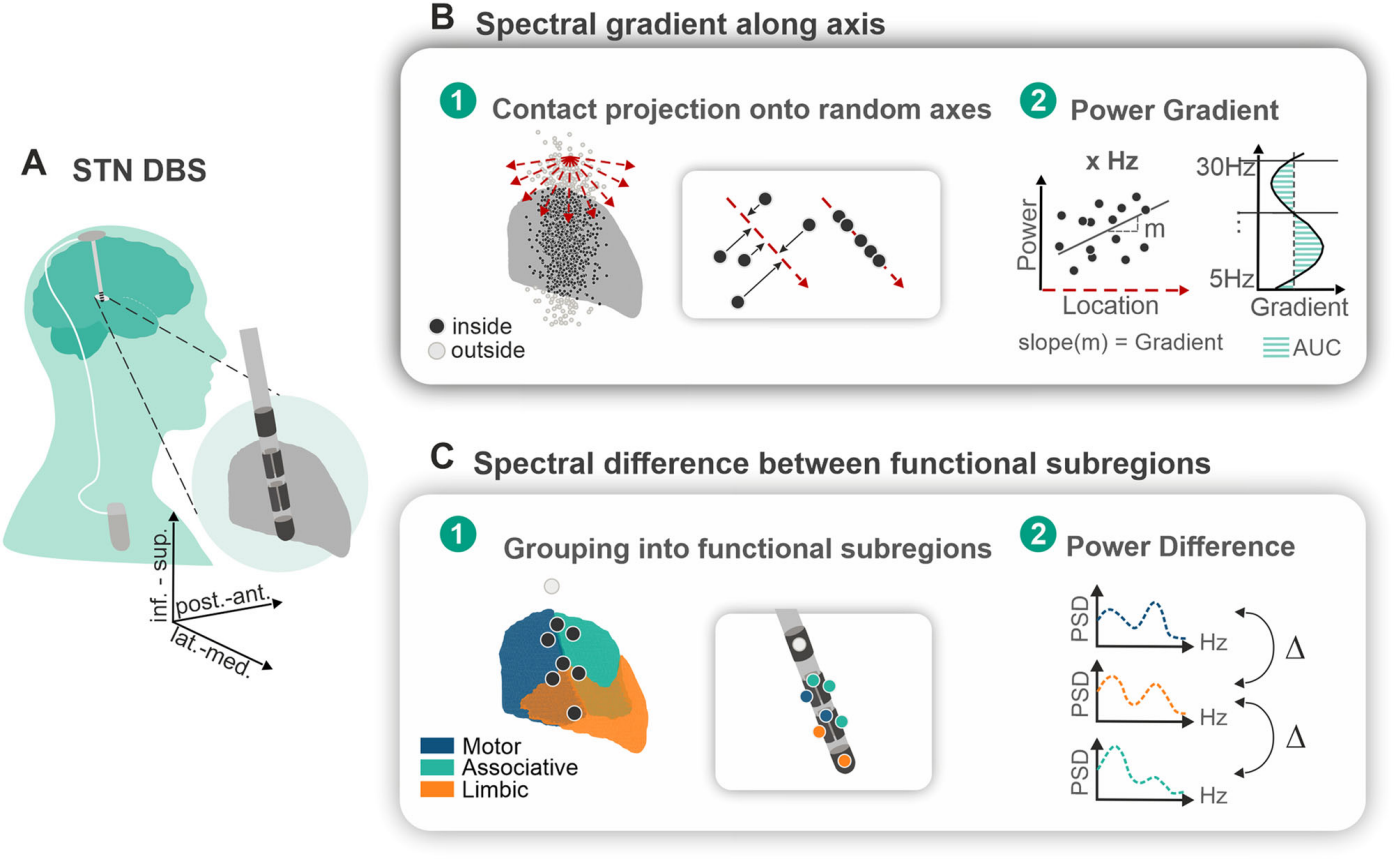
Method figure: spectral characterization of the STN. **A** Schematic illustration of the 8-contact directional DBS lead. STN schematic was created using the open-source lead-DBS toolbox^61^. **B** (1) Contacts of all subjects pooled together and projected onto the STN (black: inside STN, white: outside STN). Red, dashed arrows represent the 1000 random pluri-directional axes. The contacts within the STN were projected perpendicularly on each of the random axes. (2) For each axis, a linear regression model was fitted between the contact’s location (predictor) and their power value (outcome) for each frequency bin between 5–30 Hz. The slope of the linear fit represents the power gradient. The sum of the absolute gradient values of the single frequency bins from 5–30 Hz corresponds to the AUC and was used as index for the spectral information hold by the axis. **C** (1) Presents a schematic illustration of the tripartite subdivision of the STN (blue: motor, green: associative, orange: limbic) with the projected contacts of a DBS lead. Contacts were grouped according to their location in the different subregions of the STN. (2) The difference of the mean power spectral density of the contacts in the three functional subregions was computed for the frequencies between 5 and 30 Hz. STN subthalamic nucleus, AUC area under the curve.

**Fig. 3:**
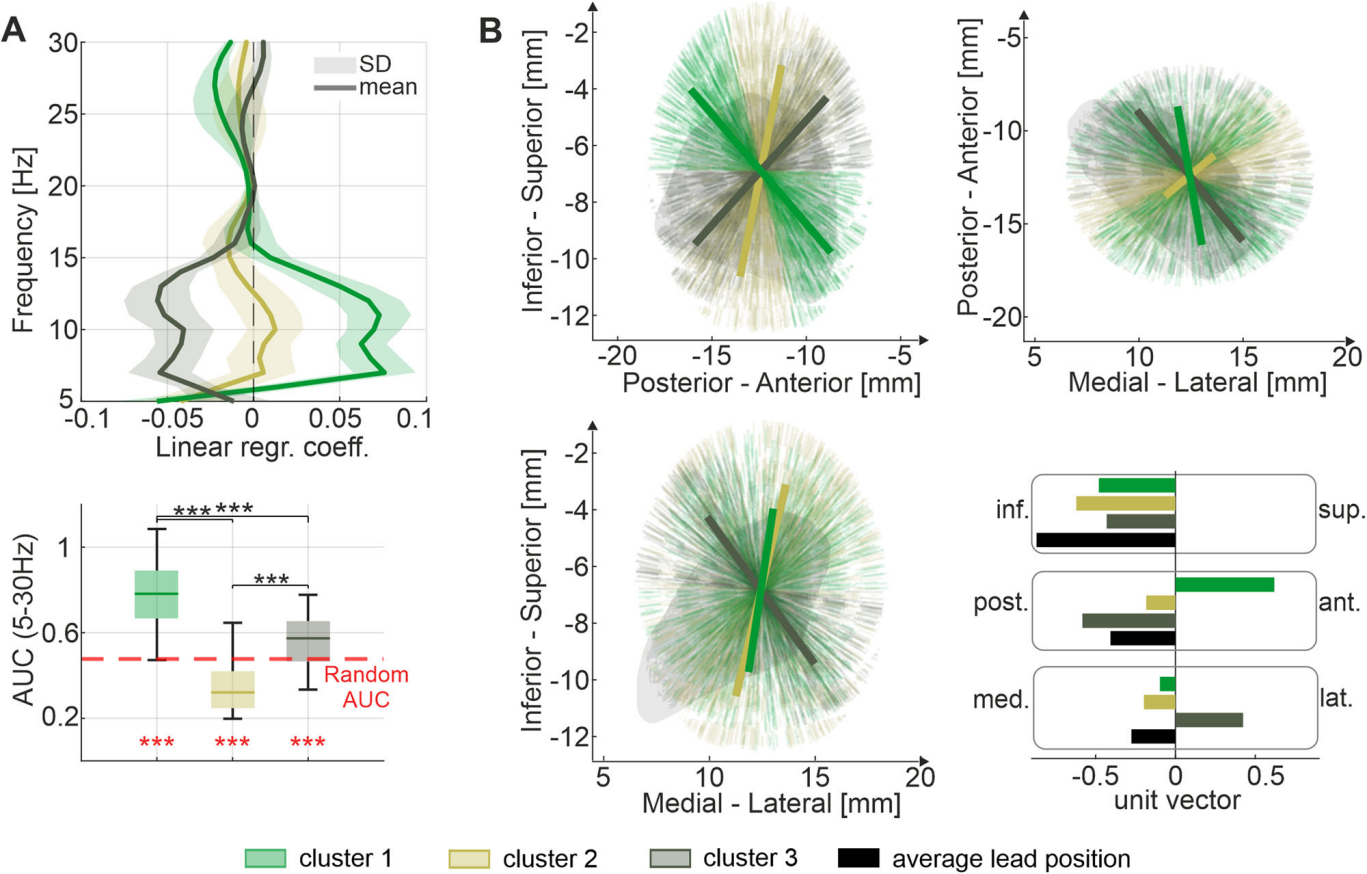
Power gradients within the STN. **A** Illustrates the three 5–30 Hz power gradient k-means clusters as mean ± SD, derived from 1000 random trajectories based on all contacts inside the STN pooled together. Note that non-standardized regression coefficients are shown. The boxplot below shows the absolute AUC of the gradient curves within the three clusters (median cluster 1 = 0.78, median cluster 2 = 0.32, median cluster 3 = 0.57). The dashed red line indicates the mean AUC by chance derived from a random surrogate distribution. All clusters had a significantly different AUC compared to the chance level and between each other (*p* < 0.001). Cluster 1 showed the highest AUC compared to the other clusters (*p* < 0.001). **B** Illustrates the respective 1000 random trajectories along the medial/lateral, the posterior/anterior and the inferior/superior axes, with the mean direction of the trajectories in a cluster in bold. The boxplot quantifies the mean directions in the three dimensions. Cluster 1 points toward inferior-medial-anterior, cluster 2 points toward inferior-medial-posterior, and cluster 3 toward inferior-lateral-posterior. As comparison, the mean trajectory of the DBS leads is illustrated in black. SD standard deviations, STN subthalamic nucleus, AUC area under the curve.

The dataset consists of a cohort of 69 patients with Parkinson’s disease with neurophysiological, anatomical, and clinical data, analysed through different pipelines (Fig. 1). The first goal of this work was to characterize the continuous spectral transitions within the anatomical STN by capturing the largest power variations (i.e. power gradients) between 5 and 30 Hz across 1000 randomly directed axes (Fig. 2). Power gradients represent the relationship between contacts’ location along one axis and their respective power values in the frequency range of interest. Using the *k*-means clustering approach, the resulting 1000 power gradients (Fig. 3A) and their corresponding axes (Fig. 3B) were grouped based on their spectral similarity. This resulted in 3 different clusters (cluster 1: 373 axes, cluster 2: 359 axes, cluster 3: 268, see Supplementary Fig. 1). The power gradients of cluster 1 and 3 show a significantly higher area under the curve (AUC) than the AUCs derived by the surrogate distribution (*p* < 0.001) (Fig. 3A). Moreover, the AUC of cluster 1 is significantly higher compared to the AUC of cluster 2 and cluster 3 (*p* < 0.001). Thus, the neurophysiological gradients related to cluster 1 holds most of the spectral information. The mean unit vector of the axes in cluster 1 is directed toward medial, anterior, inferior (*x* = −0.10, *y* = 0.61, *z* = −0.48), while the mean unit vector of cluster 2 points toward medial, posterior, inferior (*x* = −0.20, *y* = −0.18, *z* = −0.62), and the mean unit vector of cluster 3 toward lateral, posterior, inferior (*x* = 0.42, *y* = −0.58, *z* = −0.43) (Fig. 3B). The magnitude of the gradients captured by the mean axis directions of the three clusters were statistically tested against a surrogate distribution showing significant gradient values along the mean axis of cluster 1 in both the low frequency range and the beta range, while cluster 3 showed an effect in the low frequency range only (Supplementary Fig. 2). In conclusion, as the mean trajectory of cluster 1, spanning along the dorsal to ventral STN, was holding most of the spectral information for the frequency bands of interest (highest AUC), it was selected as the “preferred spectral axis”. Note that the trajectory of cluster 1 is not simply a consequence of the overall angular positions of the DBS leads, as it diverges from it by 75.6 degrees.

### Dorsal-ventral spectral and clinical gradient

**Fig. 4:**
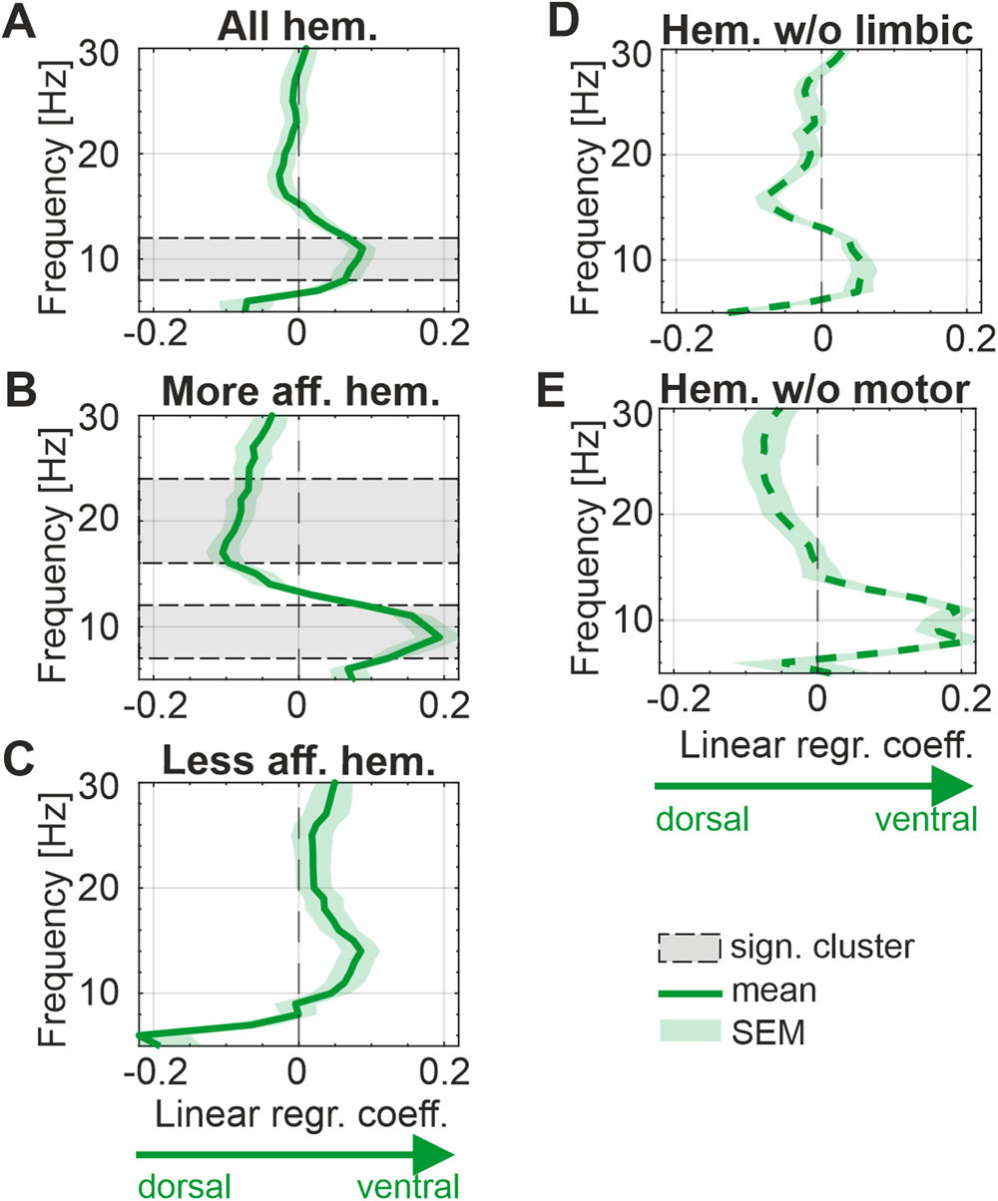
Spectral gradient along dorsal–ventral axis. **A** Shows the spectral gradients represented as linear regression coefficients from 5–30 Hz averaged across all hemispheres ± SEM. Note, higher regression coefficients toward right indicates increased power in the ventral STN, while a gradient toward left indicates increased power in the dorsal STN. The shaded area indicates a significant cluster between 8 and 12 Hz. **B** Presents the spectral gradient averaged across hemispheres contralateral to the more affected hemi-body side (±SEM). The shaded area indicates a significant cluster between 7 and 12 Hz and a second cluster between 16 and 24 Hz. **C** Presents the power gradient averaged across hemispheres contralateral to the less affected hemi-body side (±SEM), which does not evidence a significant power gradient. **D** Illustrates the average spectral gradient (±SEM) considering all hemispheres which have no contacts in the limbic STN. **E** Illustrates the average spectral gradient (±SEM) considering all hemispheres which have no contacts in the motor STN; Note, gradients were calculated considering only hemispheres with a minimum of 5 contacts inside the STN. Note that non-standardized regression coefficients are shown. SEM standard error of the mean, STN subthalamic nucleus.

In a second step, we computed the power gradient along the previously defined “preferred spectral axis” within the single hemispheres (*n* = 74) (Fig. 4). This revealed a significant positive power gradient between 8–12 Hz (*p*-value = 0.04), indicating increased alpha power toward the ventral STN (Fig. 4A). When splitting hemispheres according to the contralateral more and less affected hemi-body side, an additional cluster in the beta frequency delineates in the STN contralateral to the more affected side (*n* = 29, 16–24 Hz, *p*-value = 0.007) (Fig. 4B, Supplementary Fig. 3). The power gradient in the STN contralateral to the clinically less affected hemi-body side (*n* = 33) showed a similar but no significant trend in the alpha frequency range, and no gradient in the beta frequency range (Fig. 4C). When directly comparing the gradients between the hemispheres of the more and less affected hemi-body side within subjects with bilateral gradient calculation available, no significant cluster, but significant differences in the 14 and 15 Hz bins can be delineated (Supplementary Fig. 4). For the direct comparison of the spectral gradient between the left and right hemispheres within subjects, no difference was found (Supplementary Fig. 5).

Interestingly, the gradient indicating elevated ventral alpha power is visually preserved also in hemispheres without contacts localized in the limbic STN (*n* = 19), which did however not reach the level of significance (Fig. 4D). Similarly, also in hemispheres without contacts localized in the motor STN (*n* = 16) dorsal beta power and ventral alpha power can still be visually delineated from the gradient curve (Fig. 4E).

**Fig. 5:**
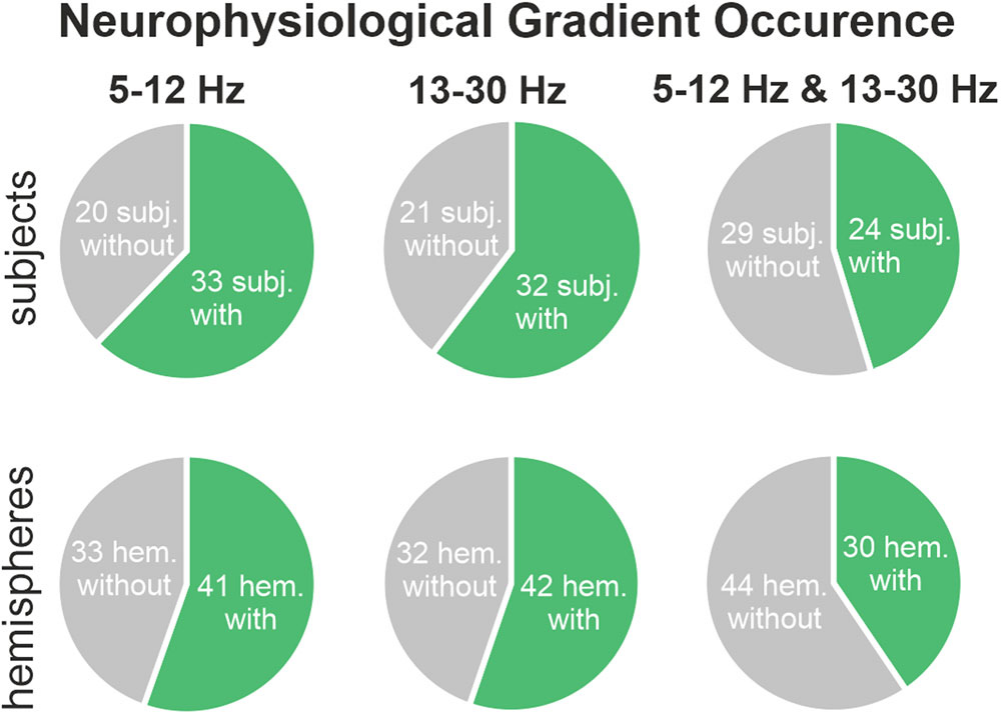
Neurophysiological gradient occurrence. Shows the fraction of occurrence of either ventral theta-alpha (5–12 Hz) power, dorsal beta (13–30 Hz) power or both; separately for two categories: occurrence of these gradients in at least one hemisphere within subject (first row) and occurrence of the gradients within hemispheres (second row).

On a semiquantitative level, the gradient analyses revealed that ventral theta-alpha power could be observed in 41 out of 74 hemispheres and dorsal beta power in 42 out of 74 hemispheres, with 30 hemispheres showing both. With regard to subjects having at least one gradient on either hemisphere, ventral theta-alpha power was observed in 33 out of 53 subjects and dorsal beta power in 32 out of 53 subjects in at least one hemisphere. Twenty-four subjects showed both, ventral theta-alpha power and dorsal beta power in at least one hemisphere (Fig. 5).

### Spectral characteristics of the STN subregions

**Fig. 6:**
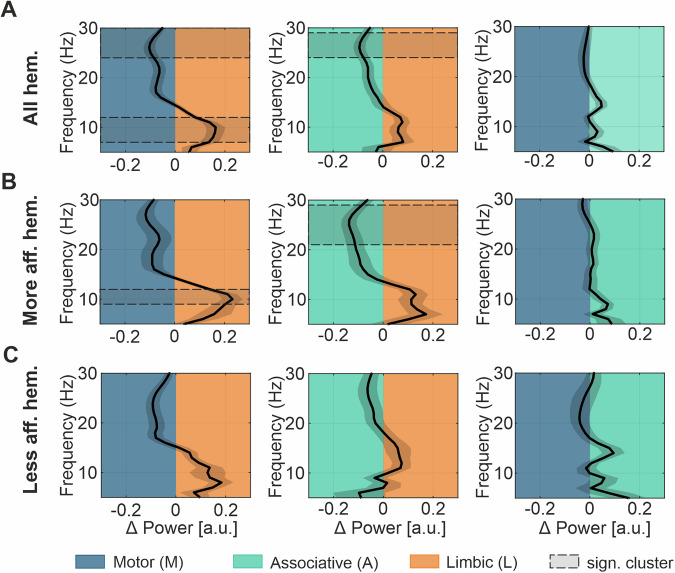
Spectral difference between functional subregions. **A** Shows the power difference between paired functional subregions from 5–30 Hz averaged across all hemispheres ± SEM. Note, the background color indicates the subregion and the deviation of the curve denotes increased power in the respective subregion. The shaded area indicates a significant cluster between 7 and 12 Hz and a second cluster between 24 and 30 Hz for the power difference between motor and limbic region. A significant cluster was also found between 24 and 29 Hz, indicating higher power in the associative compared to the limbic STN. **B** Presents the power difference averaged across hemispheres contralateral to the more affected hemi-body side. The shaded area indicates a significant cluster between 9 and 12 Hz for the power difference between limbic and motor region. A second cluster between 21 and 29 Hz indicates higher power in the associative compared to the limbic STN. **C** Presents the power differences averaged across hemispheres contralateral to the less affected hemi-body side. No significant power difference was found. SEM standard error of the mean, STN subthalamic nucleus.

To provide a more comprehensive view, we contrasted the previous representation of the spectral gradients with a classic atlas-based anatomical segregation, calculating the power distributions in the three functional subregions of the STN. Considering the entire pool of contacts of our cohort, 246 contacts are located in the motor, 270 in the associative and 117 in the limbic STN. Fifty-two hemispheres had at least one contact in both, the motor and the limbic regions, 71 hemispheres had at least one contact in the limbic and associative region and 74 hemispheres at least one contact in the motor and associative region. The power difference between subregions showed a higher sub-beta power in the limbic compared to the motor STN (cluster from 7 to 12 Hz, *p*-value = 0.03), while the latter exhibits a higher power in the beta frequency range (cluster from 24 to 30 Hz, *p*-value = 0.02) (Fig. 6A, left panel). A significantly lower beta power in the limbic region is also present when compared to the associative STN (cluster from 24 to 29 Hz, *p*-value = 0.04) (Fig. 6A, middle panel), while no spectral power differences were found between the associative and the motor STN (Fig. 6A, right panel). Similar as for the gradient analyses above, we separated the hemispheres according to the contralateral side with more and less motor impairment. Here we could still evidence the higher sub-beta power in the limbic compared to the motor STN (cluster from 9 to 12 Hz, *p*-value = 0.05), as well as the higher beta power in the associative compared to the limbic STN (cluster from 21 to 29 Hz, *p*-value = 0.02) (Fig. 6B). The sub-territories of the STN contralateral to the less affected side show no significant power difference (Fig. 6C).

### Rigidity response to DBS

**Fig. 7:**
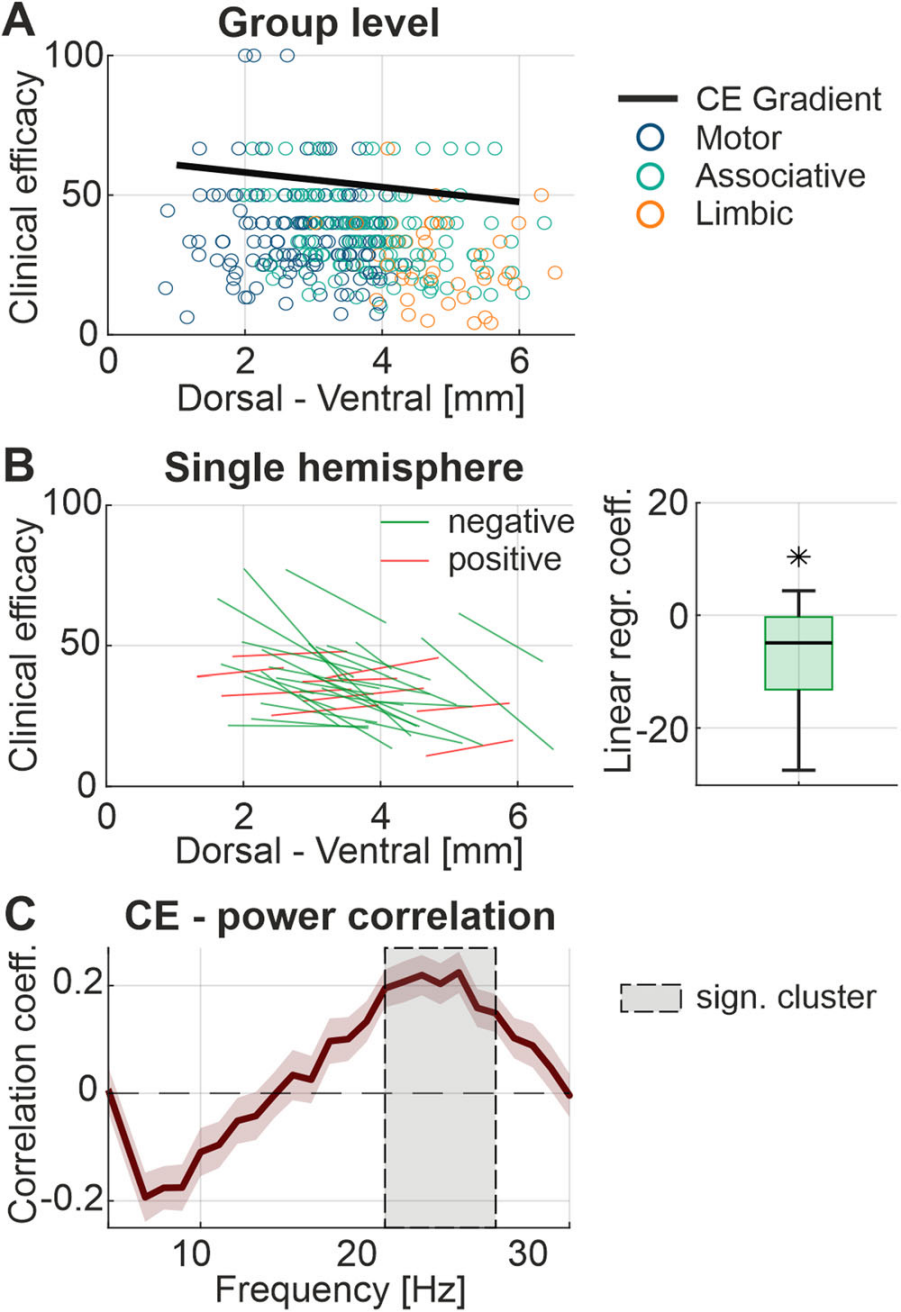
STN gradient of clinical response to DBS. **A** Shows the distribution of the entire pool of contacts inside the STN along the “preferred spectral axis” and their respective response to DBS as clinical efficacy (CE). The color indicates the STN functional subregion where each contact is located (blue: motor STN, green: associative STN, orange: limbic STN). The distribution shows a negative relationship between contact location and CE, indicating a higher CE in the dorsal STN (linear regression coefficient = −2.64, *p* < 0.001). **B** Shows the CE gradient slope for each single hemisphere with a minimum of 5 contacts inside the STN. Lines colored in green indicate a negative slope, whereas lines colored in red indicate a positive slope. The boxplot shows the CE-power gradient slope distribution with a significant negative trend (median = −4.93, *p* < 0.001). Note that non-standardized regression coefficients are shown. **C** Presents the mean correlation between CE and the power of the contacts inside the STN across hemispheres ± SEM. A significant cluster between 20 and 26 Hz indicates a positive relationship between CE and elevated beta power. CE clinical efficacy, SEM standard error of the mean, STN subthalamic nucleus.

The clinical efficacy (CE) describing the rigidity response to DBS is also changing along the “preferred spectral axis”. This was first demonstrated for all the contacts inside the STN of the entire cohort pooled together, showing a stronger motor improvement in the dorsal STN (linear regression coefficient = −2.64, *p* < 0.001) (Fig. 7A). This finding was preserved also at hemisphere level, with 27 out of 35 hemispheres showing a better rigidity response to DBS with dorsal contacts (median = −4.9, Wilcoxon signed-rank test, z = −4.21, *p* < 0.001) (Fig. 1, Fig. 7B). What are the spectral properties associated to the CE across hemispheres? Correlating CE of contacts inside the STN with the power at each frequency revealed a significant positive correlation in the beta frequency range (cluster from 20–26 Hz, *p*-value = 0.02) using the cluster-based permutation test (Fig. 7C).

### Neurophysiological correlate of apathetic symptoms

**Fig. 8:**
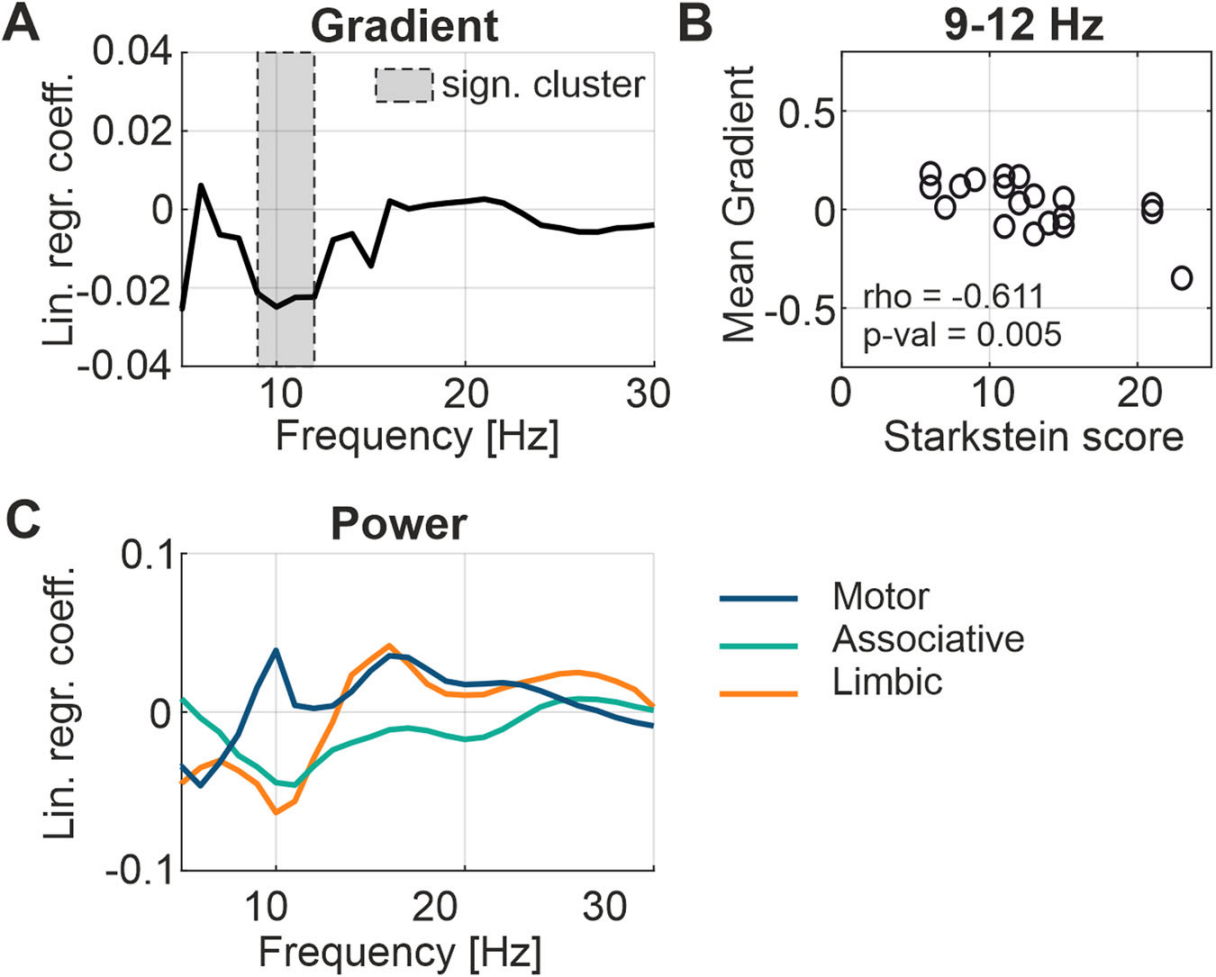
Neurophysiological correlate of apathy. **A** Illustrates the relationship between the Starkstein apathy score and the spectral gradient at each frequency bin from 5–30 Hz of 20 subjects (with a minimum of 5 contacts inside the STN in at least one hemisphere). A significant cluster between 9 and 12 Hz indicates a significant relationship between low ventral / high dorsal alpha activity and an increased apathy score. Note that non-standardized regression coefficients are shown. **B** Scatterplot between the apathy scores and the averaged 9–12 Hz power gradients. The values were significantly correlated (Spearman’s rho = −0.61, *p*-value = 0.005). **C** Shows the relationship between the Starkstein apathy score and the mean power in the functional subregions (nr. of subjects: motor = 21, associative = 25, limbic = 23) at each frequency bin between 5 and 30 Hz. The difference between the linear regression coefficient curves of the three regions did not reach significance.

Finally, we investigated the relationship between the spectral distribution in the STN and the degree of apathetic symptoms before the surgery. For this analysis, if recordings were performed from the left and right STN and both sides had a minimum of 5 contacts inside the STN, the spectral gradients of both sides were averaged prior to the analyses. Twenty-eight subjects were assessed with the Starkstein apathy scale with a mean score of 12, ranging from 5–23 (Fig. 1). We found that the spectral gradient in the alpha range was significantly, negatively related to the scores on the apathy scale (*n* = 20, significant cluster from 9–12 Hz, *p*-value = 0.04) (Fig. 8A). This means that relative low ventral compared to dorsal alpha power is linked with an increase in apathy severity. To visualize this relationship, we correlated the mean gradients between 9 and 12 Hz with the apathy scores (rho = −0.61, *p*-value = 0.005; Fig. 8B). In line with the role of the ventral STN, the power association of the STN subregions with the apathy scores showed a similar negative trend between 9 to 12 Hz for both the limbic (*n* = 23) and associative (*n* = 25), but not the motor (*n* = 21) region (Fig. 8C). The relationship between apathetic symptoms with the power of the three functional subregions did not significantly differ (see Supplementary Fig. 6). Finally, we investigated the presence of a lateralization for the relationship between apathetic symptoms with the spectral gradient between more and less affected hemi-body side (Supplementary Fig. 7) as well as between the left and right hemisphere (Supplementary Fig. 8), which both revealed no significant difference. Furthermore, to assess the impact of potential confounding factors influencing the apathy score of this cohort, we tested the relationship to the age at the exam (rho = 0.02, *p*-value = 0.94), the total UPDRS-III score (rho = 0.09, *p*-value = 0.67), the Mini-Mental State score (rho = −0.27, *p*-value = 0.24) and the Beck Depression Inventory score (rho = 0.06, *p*-value = 0.78) which all resulted in no significant relationship (Supplementary Fig. 9).

## Discussion

This work highlights the clinical and neurophysiological continuum of the motor and limbic STN in PD. Based on STN local field potential (LFP) recordings from multi-contact DBS leads in a large cohort of patients, we evidenced a preferred power gradient spanning along a dorsal-ventral axis within the STN with elevated beta power dorsally and increased alpha activity ventrally. High dorsal beta power may be indicative for motor impairment and for the clinical response of rigidity to DBS. On the contrary, we found a correlation between low ventral alpha power and a higher degree of apathetic symptoms in this cohort. This suggest that potentially a multitude of clinical-neurophysiological domains can be picked up from DBS leads implanted in the STN. As further evidence emerges, this may provide valuable guidance for optimally balancing medication and stimulation beyond motor symptom control in PD in the upcoming era of sensing-based treatment strategies.

Why to study the clinical and neurophysiological continuum of the motor-limbic STN? It is more and more recognized that the management of neuropsychiatric symptoms in PD is of great importance for the quality of life of patients^2,3,7,38–40^. Especially in the light of upcoming sensing-enabled DBS that might allow to objectively detect and monitor symptom states of patients, a comprehensive treatment approach should involve the neurophysiology of both the motor and neuropsychiatric domain^34,35^. While the beta frequency range (13–30 Hz) is best described in the context of motor symptoms in PD, some reports suggest that sub-beta frequencies from 5–12 Hz are related to impulsivity and emotional control^27–30,41–43^. Accordingly, we defined the frequency range from 5–30 Hz as the spectral band of interest in this work. To provide a continuous neurophysiological representation of this spectral range across the STN subregions, we implemented a new gradient metric. With this data-driven approach, we could evidence that the most informative spectral gradient in this frequency range spans along a dorsal-ventral axis, allocating the beta frequency activity to the dorsal and the sub-beta oscillations toward the ventral, limbic STN. While an increase of beta power in the dorsal STN is well known, this work supports the presence of higher alpha activity toward the ventral STN that so far has only been described by few studies^31,32^. What is the functional relevance of this spectral gradient present in a majority of subjects? With regard to motor control, we found that a more prominent beta frequency gradient may index the hemi-body side with a stronger motor impairment. This is likely due to a higher, yet spatially refined beta synchronization contralateral to the more affected hemi-body side^44^. Moreover, the stimulation contacts providing a better rigidity response to DBS also pick up more beta activity and are located more dorsally on the STN gradient axis, which is in line with previous results^12,32,45–48^. On the other hand, focusing on the neuropsychiatric domain, we found that a higher degree of apathetic symptoms was associated with low ventral alpha activity in the STN, which has not been described yet at the level of the basal ganglia. Given that this finding is more evident when applying the gradient metric than strictly extracting activities from atlas-defined subregions, it supports the continuous transition of clinical and spectral properties across functional subregions. Yet, we cannot differentiate whether this spectral link to apathy reflects a primarily pathological or compensatory process or whether it is secondary to other psychological deficits. In fact, although we did not observe a significant relationship of apathy with age, cognition, depression or motor performance in this cohort, potential confounds cannot be excluded. On a more general line however, evidence is accruing that sub-beta frequencies play an epiphenomenal role for neuropsychiatric symptoms. Our observation is supported by previous work showing that a high level of alpha power in the ventral STN is related to motor trait impulsivity^27^ and high-level of theta activity ON-medication may differentiate PD patients with ICDs from those without^28^. Importantly, in the present work LFPs were recorded after complete withdrawal of dopaminergic medication prior to surgery. This might have favored not only the elevated beta power in the dorsal STN, but also the detection of apathy-related activity caused by the mesolimbic denervation and the hypodopaminergic state^5^. Thus, these findings could be in synergy with the concept of a behavioral spectrum with apathy and impulsivity at the extremes, that is modulated by dopamine and with sub-beta oscillations being the corresponding neurophysiological marker. Moreover, considering the robust connection of the limbic STN to prefrontal and frontal cortical structures, it seems reasonable that a decrease in alpha synchronization would occur both cortically and subcortically^8,49–53^. In line with this are recent data showing that post-operative increase in apathy severity is associated with a reduction of 8–10 Hz functional connectivity of the bilateral prefrontal cortex with the rest of the brain during STN DBS^54^.

Although previous data suggest some functional left-right lateralization with a tendency of reversing apathy with stimulation in the left ventral STN^54–56^, we could not evidence a lateralization of the spectral relationship to apathy in our cohort. Similarly, no lateralization for the spectral link to apathy was found between hemispheres of the more and less affected hemi-body side, which is in line with the absence of a significant difference between the spectral gradients in the alpha range in the paired comparison.

What could be the practical implications of this work? Our results suggest that routinely implanted STN DBS leads may provide neurophysiological information of the functional anatomical environment that indexes not only the motor but also the limbic domain. Further research is required to confirm and expand these observations in order to fill the knowledge gap on markers for neuropsychiatric symptoms. Moreover, one needs to be careful with directly transferring knowledge acquired from retrospective research data into clinical use cases, specifically as the response of the biomarker to medication or chronic stimulation is not yet understood. Once this is the case, clinical developments could include brain sensing to help identifying patients at risk of developing neuropsychiatric symptoms such as impulsivity or postoperative apathy and to inform the clinician to adjust stimulation or medication accordingly. Moreover, it may inform sensing-assisted DBS programming for selecting the preferred stimulation site or avoiding the one leading to undesired stimulation side-effects based on the neurophysiological environment picked up from the DBS contacts^39,40,45,57,58^. With regard to adaptive closed-loop DBS, robust non-motor symptom biomarkers may support future efforts working toward integrated and multimodal approaches to comprehensively monitor and potentially control neuropsychiatric- in addition to motor symptoms in real time in PD^35^.

One limitation of this study is that the pre-operative motor and neuropsychiatric scores as well as the postoperative response to DBS have not been assessed at the same time as the LFP measurement. Although there is sufficient evidence that spectral properties and symptom propensities can be related to each other despite being assessed at different time points^12,27^, this might have still reduced the effect size of our results. Additionally, patients may suffer distress during awake surgery and its impact on limbic signaling is unknown. Hence, dedicated acute and chronic experimental set-ups on larger cohorts should be envisioned to predict changes in apathy and impulsivity in response to different levels of dopaminergic medication. Note, the allocation of contacts to the STN subregions may partially be inaccurate due to distortion after anatomical normalization of the STN in the MNI space, while the region independent gradient analyses are likely to be less affected by this. Moreover, it must be stated that the mechanisms giving rise to the magnitude and shape of the gradients are not entirely understood and need to be further characterized in larger cohorts, different target regions and conditions in order to bring them into the biological context of brain networks.

Finally, the clinical-neurophysiological motor-limbic continuum detectable within the STN encourages further research to explore and refine not only motor, but also neuropsychiatric symptom biomarkers. This is of particular interest in the currently evolving era of sensing-guided neuromodulation, which may facilitate integrated treatment approaches to optimally balance medication and stimulation for controlling multimodal symptoms in PD.

## Methods

### Patients and surgery

This single-center study was approved by the local ethics committee of Bern (2017-00551) and adhered to the ethical standards of the Declaration of Helsinki. As required by the ethics protocol all subjects signed the general consent.

PD patients included in this study underwent STN DBS surgery at the University Hospital in Bern from December 2015 to December 2021 (Fig. 1). Out of 69 patients in total, 41 were males and 28 females with an average age of 62.1 (range 33 to 77), an average disease duration of 9.6 years (range 3–24) and a total MDS-UPDRS-III (Movement Disorders Society—Unified Parkinson’s disease Rating Scale part 3) score of 39.2 pts (range 19–70) OFF-medication. All patients were implanted with the Boston Vercise Cartesia directional leads (Boston Scientific Cartesia, Marlborough, MA) (Fig. 2A). The DBS target was identified on the T2-sequence of the preoperative 3-T magnetic resonance imaging (MRI) and preoperative stereotactic computed tomography (CT) scans (Leksell G frame) using Brainlab Elements software (Brainlab AG, Munich, Germany)^59^. Intraoperative targeting was optimized using microelectrode recording and selective test stimulation.

### LFP recordings and signal processing

LFPs were recorded intraoperatively from 128 out of 138 hemispheres (Fig. 1) with patients awake and at rest. The recordings were conducted from all eight contacts in a monopolar setting for 1–3 min after final placement of the DBS lead. In 49 patients LFPs were recorded using the TMSi-Porti amplifier (Twente Medical Systems International, Oldenzaal, Netherlands) with a common average referencing and a sampling rate of 2048 Hz. In 20 patients LFPs were recorded using the Inomed (INOMED Inc., Tenningen, Germany) system with the cannula as common reference and a sampling rate of 2000 Hz. Data were visually inspected in MATLAB R2020b (Natick, Massachusetts: The MathWorks Inc.) and periods with gross artefacts occurring in all channels simultaneously were removed. Dopaminergic medication was withdrawn before surgery (levodopa: 12 h, dopamine agonists: 48 h). After pre-processing and artefact removal performed in line with our previous work^12^, signals with a mean duration of 82 s (range: 14–171) were available for further analyses. The signals were down-sampled to 800 Hz, high-pass filtered at 0.5 Hz and notch filtered at 50 Hz. Then signals were decomposed into frequency components of 1 Hz resolution using a Wavelet transform (ft_specest_wavelet- Morlet Wavelet, width = 10, gwidth = 5; Fieldtrip, Donders Institute for Brain, Cognition and Behaviour, 2010). The frequency ranges of interest were selected in accordance with previous literature on motor and neuropsychiatric related frequencies: sub-beta range from 5–12 Hz and beta range from 13–30 Hz^14,16,18,27,46,60^. In order to increase the comparability of signals between contacts and across hemispheres, the power spectral density of each contact was normalized by subtracting its mean over the reference frequency range (5–45 Hz and 55–80 Hz) and dividing by its SD over the same frequency range.

### Localization of DBS contacts

The postoperative localization of DBS contacts was performed using the Lead-DBS MATLAB toolbox (version 2.6)^61^. Preoperative MRI and postoperative CT scans were co-registered and normalized to the Montreal Neurological Institute (MNI) space (MNI152 NLIN 2009b). The DISTAL atlas was used to visualize and localize contact’s position with respect to motor, associative and limbic subregions, informed by cortical STN structural connectivity^8,10,11^. The contact locations from the left STN were projected onto the right STN using a nonlinear flip function. The MATLAB function *intriangulation* was used to detect contacts inside the STN. The electrode trajectory and position were reconstructed semi-automatically using the Precise and Convenient Electrode Reconstruction for Deep Brain Stimulation (PaCER), and then manually corrected if necessary^62^. The orientation of the electrode was determined with DiODe using the artefact of orientation marker^63^. The potential mismatch of the effective and the surgically intended orientation of the DBS lead in this cohort was discussed in a previous work^12^. The in-built MATLAB function *intriangulation* was used to detect contacts inside the STN. Out of the total cohort of contacts (*n* = 993), 59% were detected inside the STN.

### Spectral distribution in the STN

To determine the continuous power distribution of the frequency range of interest within the STN, a power gradient method has been established (Fig. 2B). In a first step, a pool of 1000 axes with random angles was generated, all of them with a unit vector *z*-value ranging from 0 to -1 (i.e. pointing toward inferior) (in order to avoid mirroring of the axes). The location of the entire cohort’s contacts inside the STN were orthogonally projected on each of these axes (Fig. 2B, 1). The relationship between the contact location on the axis, measured in millimeters, and its normalized power at the frequencies ranging from 5–30 Hz was determined using a robust linear regression model (MATLAB function *fitlm* with a bisquare weighting function) to account for potential outliers. This method provides reliable estimates even in the presence of extreme values by reducing their influence on the overall fit (Fig. 2B, 2). The resulting curve of non-standardized regression coefficients over the frequencies of interest represent the “power gradient” of the single axes. Secondly, all the 1000 random power gradient curves were grouped into clusters using the k-means clustering method. K-means clustering is a machine learning algorithm that divides a dataset into k distinct, non-overlapping clusters based on similarity, aiming to minimize the within-cluster variance. The optimal number of clusters was identified using the elbow method. This involves plotting the sum of squared distances from data points to their respective cluster centroids against the number of clusters, and visually determining the point where the rate of decrease in within-cluster variance drastically slows down, known as the “elbow point”. Next, we determined the cluster holding the most spectral information. For this we computed the AUC of the respective power gradients, which corresponds to the sum of the absolute gradient values of the frequency bins from 5–30 Hz. First, the AUC of the clusters was compared to a surrogate distribution of AUCs received by randomly shuffling of the contacts’ power values (1000 permutations), while keeping number and location of contacts constant. Second, the cluster with the highest AUC holding most of the spectral information was determined and its mean axis direction termed the “preferred spectral axis”. This axis was used for the gradient analyses at hemisphere level. To assure the regression models are robust, only hemispheres with a minimum of 5 contacts inside the STN were included in this analysis. Additionally, to compare the results to the more classic atlas-based anatomical segregation, contacts were assigned to the corresponding motor-, associative-, and limbic region depending on their x-,y-,z-coordinates in the normalized space (Fig. 2C, 1). For each hemisphere, the average power of the contacts located in the three regions was calculated and contrasted between the regions (Fig. 2C, 2).

### Pre-operative motor and neuropsychiatric assessment

Three months (±2.5 months) prior to surgery, all patients underwent a series of motor and neuropsychiatric assessments (Fig. 1). The motor impairment in the OFF-medication state was assessed using the MDS-UPDRS-III in all subjects^64^. For a sub-analysis this was then further split into the more and less affected side including all hemi-body items (bradykinesia, rigidity, and tremor items). Apathy was evaluated by the self-assessed Starkstein questionnaire^65^ in 28 patients. Since the Starkstein questionnaire was included in our clinical routine only later in time, it was not assessed in the entire retrospective cohort pre-operatively.

### Assessment of rigidity response to DBS

A monopolar contact review was performed 5 months (±1.5 months) after surgery by specialized staff with the patient in the medication OFF state. The reduction of upper limb rigidity was used to determine the clinical response to DBS, as this is the clinical sign considered the most sensitive for indexing DBS response during the monopolar contact review^66^. The effect threshold, representing the current required to fully alleviate rigidity or to achieve optimal improvement, was determined by incrementally increasing the amplitude in 0.5 mA steps, while keeping the stimulation frequency and pulse width constant at 130 Hz and 60 μs, respectively. The clinical efficacy (CE) was calculated for hemispheres with at least one point in MDS-UPDRS-III upper-limb rigidity at baseline using the formula (1) described in previous studies^12,45,58^:

1 $${Clinical\; efficacy}\left({CE}\right)=\,\frac{100\,\times \,({rigidity\; at\; baseline}-{rigidity\; at\; effect\; threshold})}{{rigidity\; at\; baseline}\times {current\; at\; effect\; theshold}}$$

A CE gradient was computed along the “preferred spectral axis” by fitting a robust linear regression model to all contacts’ axis location (independent variable) and their CE values (dependent variable). The CE gradient was calculated based on all contacts pooled together as well as for each hemisphere with a minimum of 5 contacts localized inside the STN. Note that the CE gradient values represent the non-standardized regression coefficients.

### Statistical analysis

All statistical analyses were performed using MATLAB (version R2022a; MathWorks). Results are presented as mean ± standard error of the mean (SEM), unless otherwise stated.

The AUCs of the power gradient clusters were compared to a surrogate distribution of randomly generates AUC’s using a Wilcoxon rank sum test. A Kruskal-Wallis test was applied to compare the AUCs between the clusters. To control for multiple comparison, *p*-values were FDR (false discovery rate) corrected. A cluster-based permutation was performed to assess the significance of the spectral gradients within hemispheres, the spectral gradient difference between the hemispheres of the more and less affected hemi-body side, between the left and right hemispheres, as well as the power difference between functional sub-regions within hemispheres across the frequency range between 5 and 30 Hz. The sign of the gradient values and the differences respectively were randomly permuted 1000 times for a subset of hemispheres. For each frequency point, the z-statistic (of the actual mean gradient / gradient difference) was computed based on the distribution of the 1000 permutations. The supra-threshold clusters (pre-cluster threshold: *p* < 0.05) were determined for each permutation, and the sum of the z-statistics within these clusters was stored to form a distribution of the largest supra-threshold-cluster values. Finally, the 95th percentile of this distribution served as statistical threshold for the map of the actual z-statistics of the real difference^67^. With this approach, we control for the family-wise error rate by assessing the significance of clusters as a whole rather than at individual points. For a sub-analysis, the difference in spectral gradients between sides has been additionally tested for each frequency bin using a Wilcoxon signed rank test. The distribution of CE gradient values within each hemisphere was tested against zero using a nonparametric Wilcoxon signed-rank test after assessing the data distribution with the Lilliefors test for normality. For each hemisphere, the relationship between the CE values and the power values at the frequency bins between 5–30 Hz was assessed using the Spearman’s correlation coefficient and its distribution tested for statistical significance using the cluster-based permutation procedure described above. Before assessing the relationship between the apathy score and the spectral data, left and right spectral data were averaged within each subject. Then, a robust linear regression model was fitted at each frequency bin from 5–30 Hz with the apathy score as predictor variable. This method allows to account for potential outliers. Its non-standardized linear regression coefficients (slopes) over the defined frequency range were tested for significance using the cluster-based permutation procedure. Here, a permutation distribution was derived by shuffling the order of the scores 1000 times and calculating the linear relationship with the spectral gradients for each permutation. At the frequency bins within the significant cluster, the spectral gradient values were averaged and correlated (Spearman) with the apathy score after testing and removal of potential outliers. A robust linear regression model was also fitted to assess the relationship of the power at each frequency bin from 5–30 Hz in each functional subregion of the STN with the apathy score as predictor variable. The resulting three regression coefficient curves over the frequency range of interest were compared using a bootstrap method (1000 cycles) to determine the 95th confidence interval of the differences at each frequency bin. This method was also applied to test the difference in the relationship of the apathy score with the gradients of the hemispheres contralateral to the more and less affected hemi-body side as well as of the left and the right hemisphere.

## Supplementary information

Supplementary material

## Data Availability

The data are available upon reasonable request to the corresponding author, gerd.tinkhauser@insel.ch.

## Code availability

The codes are available upon reasonable request to the corresponding author, gerd.tinkhauser@insel.ch.

## References

[1] Bloem, B. R., Okun, M. S. & Klein, C. Parkinsonas disease. *Lancet* **397**, 2284–2303 (2021). 33848468 10.1016/S0140-6736(21)00218-X

[2] Pagonabarraga, J., Kulisevsky, J., Strafella, A. P. & Krack, P. *Apathy in Parkinson’s Disease: Clinical Features, Neural Substrates, Diagnosis, and Treatment*. www.thelancet.com/neurology (2015). 10.1016/S1474-4422(15)00019-8 25895932

[3] Lhommée, E. et al. Subthalamic stimulation in Parkinson’s disease: restoring the balance of motivated behaviours. *Brain* **135**, 1463–1477 (2012). 22508959 10.1093/brain/aws078

[4] Czernecki, V. et al. Apathy following subthalamic stimulation in Parkinson disease: a dopamine responsive symptom. *Mov. Disord.* **23**, 964–969 (2008). 18398913 10.1002/mds.21949

[5] Thobois, S. et al. Behavioral disorders in Parkinson’s disease: rom pathophysiology to the mastery of dopaminergic treatment. *Rev. Neurol. (Paris)* **166**, 816–821 (2010). 20739041 10.1016/j.neurol.2010.07.006

[6] Martinez-Martin, P., Rodriguez-Blazquez, C., Kurtis, M. M. & Chaudhuri, K. R. The impact of non-motor symptoms on health-related quality of life of patients with Parkinson’s disease. *Mov. Disord.* **26**, 399–406 (2011). 21264941 10.1002/mds.23462

[7] Debove, I. et al. Management of impulse control and related disorders in Parkinson’s disease: an expert consensus. *Mov. Disord.* **39**, 235–248 (2024). 38234035 10.1002/mds.29700

[8] Haynes, W. I. A. & Haber, S. N. The organization of prefrontal-subthalamic inputs in primates provides an anatomical substrate for both functional specificity and integration: Implications for basal ganglia models and deep brain stimulation. *J. Neurosci.* **33**, 4804–4814 (2013). 23486951 10.1523/JNEUROSCI.4674-12.2013 PMC3755746

[9] Mallet, L. et al. *Stimulation of Subterritories of the Subthalamic Nucleus Reveals Its Role in the Integration of the Emotional and Motor Aspects of Behavior*. www.pnas.org/cgi/content/full/ (2007). 10.1073/pnas.0610849104 PMC1965569 17556546

[10] Ewert, S. et al. Toward defining deep brain stimulation targets in MNI space: A subcortical atlas based on multimodal MRI, histology and structural connectivity. *NeuroImage* **170**, 271–282 (2018). 28536045 10.1016/j.neuroimage.2017.05.015

[11] Obeso, J. A. et al. Functional organization of the basal ganglia: therapeutic implications for Parkinson’s disease. *Mov. Disord.* **3**, S548–59 (2008). 10.1002/mds.22062 18781672

[12] Averna, A. et al. Spectral topography of the subthalamic nucleus to inform next-generation deep brain stimulation. *Mov. Disord.* **38**, 818–830 (2023). 36987385 10.1002/mds.29381 PMC7615852

[13] Rodriguez-Rojas, R. et al. Functional topography of the human subthalamic nucleus: relevance for subthalamotomy in Parkinson’s disease. *Mov. Disord.* **37**, 279–290 (2022). 34859498 10.1002/mds.28862

[14] Brown, P. et al. Dopamine dependency of oscillations between subthalamic nucleus and pallidum in Parkinson’s disease. *J. Neurosci.* **21**, 1033–1038 (2001). 11157088 10.1523/JNEUROSCI.21-03-01033.2001 PMC6762327

[15] Khawaldeh, S. et al. Balance between competing spectral states in subthalamic nucleus is linked to motor impairment in Parkinson’s disease. *Brain* **145**, 237–250 (2022). 34264308 10.1093/brain/awab264 PMC8967096

[16] Kühn, A. A., Kupsch, A., Schneider, G. & Brown, P. Reduction in subthalamic 8–35 Hz oscillatory activity correlates with clinical improvement in Parkinson’s disease. *Eur. J. Neurosci.* **23**, 1956–1960 (2006). 16623853 10.1111/j.1460-9568.2006.04717.x

[17] Neumann, W. J. et al. Subthalamic synchronized oscillatory activity correlates with motor impairment in patients with Parkinson’s disease. *Mov. Disord.* **31**, 1748–1751 (2016). 27548068 10.1002/mds.26759 PMC5120686

[18] Tinkhauser, G. et al. The cumulative effect of transient synchrony states on motor performance in parkinson⇔s disease. *J. Neurosci.* **40**, 1571–1580 (2020). 31919131 10.1523/JNEUROSCI.1975-19.2019 PMC7044725

[19] Beudel, M. et al. Oscillatory beta power correlates with Akinesia‐Rigidity in the Parkinsonian subthalamic nucleus. *Mov. Disord.* **32**, 174–175 (2017). 27859589 10.1002/mds.26860

[20] Bange, M. et al. Subthalamic stimulation modulates context-dependent effects of beta bursts during fine motor control. *Nat. Commun.* **15**, 3166 (2024). 38605062 10.1038/s41467-024-47555-3 PMC11009405

[21] Wilkins, K. B. et al. Bradykinesia and its progression are related to interhemispheric beta coherence. *Ann Neurol.* **93**, 1029–1039 (2023). 36641645 10.1002/ana.26605 PMC10191890

[22] Feldmann, L. K. et al. Subthalamic beta band suppression reflects effective neuromodulation in chronic recordings. *Eur. J. Neurol.* **28**, 2372–2377 (2021). 33675144 10.1111/ene.14801

[23] Tinkhauser, G. et al. Beta burst dynamics in Parkinson’s disease OFF and ON dopaminergic medication. *Brain* **140**, 2968–2981 (2017). 29053865 10.1093/brain/awx252 PMC5667742

[24] Lofredi, R. et al. Beta bursts during continuous movements accompany the velocity decrement in Parkinson’s disease patients. *Neurobiol. Disord.* **127**, 462–471 (2019). 10.1016/j.nbd.2019.03.013 PMC6520224 30898668

[25] Priori, A. et al. Rhythm-specific pharmacological modulation of subthalamic activity in Parkinson’s disease. *Exp. Neurol.* **189**, 369–379 (2004). 15380487 10.1016/j.expneurol.2004.06.001

[26] Yeh, C. H. et al. Waveform changes with the evolution of beta bursts in the human subthalamic nucleus. *Clin. Neurophysiol.* **131**, 2086–2099 (2020). 32682236 10.1016/j.clinph.2020.05.035 PMC7115847

[27] Ricciardi, L. et al. Neurophysiological correlates of trait Impulsivity in Parkinson’s disease. *Mov. Disord.* **36**, 2126–2135 (2021). 33982824 10.1002/mds.28625 PMC7611688

[28] Rodriguez-Oroz, M. C. et al. Involvement of the subthalamic nucleus in impulse control disorders associated with Parkinson’s disease. *Brain* **134**, 36–49 (2011). 21059746 10.1093/brain/awq301

[29] Ricciardi, L., Apps, M. & Little, S. Uncovering the neurophysiology of mood, motivation and behavioral symptoms in Parkinson’s disease through intracranial recordings. *NPJ Parkinsons Dis.* **9**, 136 (2023). 37735477 10.1038/s41531-023-00567-0 PMC10514046

[30] Eisinger, R. S., Urdaneta, M. E., Foote, K. D., Okun, M. S. & Gunduz, A. Non-motor characterization of the Basal Ganglia: evidence from human and non-human primate electrophysiology. *Front. Neurosci.* **12**, 385 (2018). 30026679 10.3389/fnins.2018.00385 PMC6041403

[31] Rappel, P. et al. Theta‐alpha oscillations characterize emotional subregion in the human ventral subthalamic nucleus. *Mov. Dis.* **35**, 337–343 (2020). 10.1002/mds.27910 31758821

[32] Horn, A., Neumann, W. J., Degen, K., Schneider, G. H. & Kühn, A. A. Toward an electrophysiological “Sweet spot” for deep brain stimulation in the subthalamic nucleus. *Hum. Brain Mapp.* **38**, 3377–3390 (2017). 28390148 10.1002/hbm.23594 PMC6867148

[33] Beudel, M. & Brown, P. Adaptive deep brain stimulation in Parkinson’s disease. *Parkinsonism Relat. Disord.* **22**, S123–S126 (2016). 26411502 10.1016/j.parkreldis.2015.09.028 PMC4671979

[34] Neumann, W., Gilron, R., Little, S. & Tinkhauser, G. Adaptive deep brain stimulation: from experimental evidence toward practical implementation. *Mov. Disord.* **38**, 937–948 (2023). 37148553 10.1002/mds.29415

[35] Tinkhauser, G. & Moraud, E. M. Controlling clinical states governed by different temporal dynamics with closed-loop deep brain stimulation: a principled framework. *Front. Neurosci.* **15**, 734186 (2021). 10.3389/fnins.2021.734186 PMC8632004 34858126

[36] Alva, L. et al. Clinical neurophysiological interrogation of motor slowing: critical step towards tuning adaptive deep brain stimulation. *Clin. Neurophysiol.* **152**, 43–56 (2023). 37285747 10.1016/j.clinph.2023.04.013 PMC7615935

[37] Groppa, S. et al. Perspectives of implementation of closed-loop deep brain stimulation: from neurological to psychiatric disorders. *Stereot. Funct. Neurosurg.* **102**, 40–54 (2024). 10.1159/000535114 38086346

[38] Castrioto, A., Lhommée, E., Moro, E. & Krack, P. Mood and behavioural effects of subthalamic stimulation in Parkinson's disease. *Lancet Neurol.* **13**, 287–305 (2014). 24556007 10.1016/S1474-4422(13)70294-1

[39] Prange, S. et al. Limbic stimulation drives mania in STN-DBS in Parkinson disease: a prospective study. *Ann Neurol.* **92**, 411–417 (2022). 35703252 10.1002/ana.26434

[40] Zoon, T. J. C. et al. Apathy induced by subthalamic nucleus deep brain stimulation in Parkinson’s disease: A meta‐analysis. *Mov. Disord.* **36**, 317–326 (2021). 33331023 10.1002/mds.28390 PMC7986158

[41] Eitan, R. et al. Asymmetric right/left encoding of emotions in the human subthalamic nucleus. *Front. Syst. Neurosci.* **7**, 69 (2013). 10.3389/fnsys.2013.00069 PMC3810611 24194703

[42] Kühn, A. A. et al. *Activation of the Subthalamic Region during Emotional Processing in Parkinson Disease*. www.neurology.org (2005). 10.1212/01.wnl.0000174438.78399.bc 16157903

[43] Brücke, C. et al. The subthalamic region is activated during valence-related emotional processing in patients with Parkinson’s disease. *Eur. J. Neurosci.* **26**, 767–774 (2007). 17686048 10.1111/j.1460-9568.2007.05683.x

[44] Tinkhauser, G. et al. Beta burst coupling across the motor circuit in Parkinson’s disease. *Neurobiol. Disord.* **117**, 217–225 (2018). 10.1016/j.nbd.2018.06.007 PMC6054304 29909050

[45] Tinkhauser, G. et al. Directional local field potentials: a tool to optimize deep brain stimulation. *Mov. Disord.* **33**, 159–164 (2018). 29150884 10.1002/mds.27215 PMC5768242

[46] Zaidel, A., Spivak, A., Grieb, B., Bergman, H. & Israel, Z. Subthalamic span of β oscillations predicts deep brain stimulation efficacy for patients with Parkinson’s disease. *Brain* **133**, 2007–2021 (2010). 20534648 10.1093/brain/awq144

[47] Herzog, J. et al. Most effective stimulation site in subthalamic deep brain stimulation for Parkinson’s disease. *Mov. Disord.* **19**, 1050–1054 (2004). 15372594 10.1002/mds.20056

[48] Pollo, C. et al. Localization of electrodes in the subthalamic nucleus on magnetic resonance imaging. *J. Neurosurg.* **106**, 36–44 (2007). 10.3171/jns.2007.106.1.36 17240554

[49] Irmen, F. et al. Left prefrontal connectivity links subthalamic simulation with depressive symptoms. *Ann. Neurol.* **87**, 962–975 (2020). 32239535 10.1002/ana.25734

[50] van Wijk, B. C. M. et al. Functional connectivity maps of theta/alpha and beta coherence within the subthalamic nucleus region. *Neuroimage* **257**, 119320 (2022). 10.1016/j.neuroimage.2022.119320 35580809

[51] Mosher, C. P., Mamelak, A. N., Malekmohammadi, M., Pouratian, N. & Rutishauser, U. Distinct roles of dorsal and ventral subthalamic neurons in action selection and cancellation. *Neuron* **109**, 869–881.e6 (2021). 33482087 10.1016/j.neuron.2020.12.025 PMC7933114

[52] Hirschmann, J. et al. Distinct oscillatory STN-cortical loops revealed by simultaneous MEG and local field potential recordings in patients with Parkinson’s disease. *Neuroimage* **55**, 1159–1168 (2011). 21122819 10.1016/j.neuroimage.2010.11.063

[53] Muthuraman, M. et al. Effects of DBS in Parkinsonian patients depend on the structural integrity of frontal cortex. *Sci. Rep.* **7**, 43571 (2017). 10.1038/srep43571 PMC5337928 28262813

[54] Boon, L. I. et al. Structural and functional correlates of subthalamic deep brain stimulation-induced apathy in Parkinson’s disease. *Brain Stimul.* **14**, 192–201 (2021). 33385593 10.1016/j.brs.2020.12.008

[55] Eisenstein, S. A. et al. Functional anatomy of subthalamic nucleus stimulation in Parkinson disease. *Ann. Neurol.* **76**, 279–295 (2014). 24953991 10.1002/ana.24204 PMC4172323

[56] Béreau, M. et al. Motivational and cognitive predictors of apathy after subthalamic nucleus stimulation in Parkinson’s disease. *Brain* **147**, 472–485 (2024). 37787488 10.1093/brain/awad324

[57] Santin, M. des N. et al. Impact of subthalamic deep brain stimulation on impulse control disorders in Parkinson’s disease: a prospective study. *Mov. Disord.* **36**, 750–757 (2021). 33022101 10.1002/mds.28320

[58] Shah, A. et al. Combining multimodal biomarkers to guide deep brain stimulation programming in Parkinson disease. *Neuromodulation* **26**, 320–332 (2022). 10.1016/j.neurom.2022.01.017 PMC7614142 35219571

[59] Nguyen, T. A. K. et al. Directional stimulation of subthalamic nucleus sweet spot predicts clinical efficacy: proof of concept. *Brain Stimul.* **12**, 1127–1134 (2019). 31130498 10.1016/j.brs.2019.05.001

[60] Huebl, J. et al. Modulation of subthalamic alpha activity to emotional stimuli correlates with depressive symptoms in Parkinson’s disease1. *Mov. Disord.* **26**, 477–483 (2011). 21287598 10.1002/mds.23515

[61] Horn, A. & Kühn, A. A. Lead-DBS: A toolbox for deep brain stimulation electrode localizations and visualizations. *Neuroimage* **107**, 127–135 (2015). 25498389 10.1016/j.neuroimage.2014.12.002

[62] Husch, A., Petersen, M. V., Gemmar, P., Goncalves, J. & Hertel, F. PaCER - A fully automated method for electrode trajectory and contact reconstruction in deep brain stimulation. *Neuroimage Clin.* **17**, 80–89 (2018). 29062684 10.1016/j.nicl.2017.10.004 PMC5645007

[63] Hellerbach, A. et al. DiODe: Directional orientation detection of segmented deep brain stimulation leads: a sequential algorithm based on CT imaging. *Stereotact. Funct. Neurosurg.* **96**, 335–341 (2018). 30481772 10.1159/000494738

[64] Goetz, C. G. et al. Movement disorder society-sponsored revision of the unified Parkinson’s disease rating scale (MDS-UPDRS): scale presentation and clinimetric testing results. *Mov. Disord.* **23**, 2129–2170 (2008). 19025984 10.1002/mds.22340

[65] Starkstein, S. E. et al. Reliability, validity, and clinical correlates of apathy in Parkinson’s disease. *J. Neuropsychiatry Clin. Neurosci.* **4**, 134–139 (1992). 1627973 10.1176/jnp.4.2.134

[66] Volkmann, J., Moro, E. & Pahwa, R. Basic algorithms for the programming of deep brain stimulation in Parkinson’s disease. *Mov. Disord.* **21**, S284–9 (2006). 10.1002/mds.20961 16810675

[67] Maris, E. & Oostenveld, R. Nonparametric statistical testing of EEG- and MEG-data. *J. Neurosci. Methods* **164**, 177–190 (2007). 17517438 10.1016/j.jneumeth.2007.03.024

