# Supplementary material for: Neurophysiological gradient in the Parkinsonian subthalamic nucleus as a marker for motor symptoms and apathy
Source: NPJ Parkinsons Dis. 2025 Jan 3;11:4. doi: 10.1038/s41531-024-00848-2 (PMC11698975; doi:10.1038/s41531-024-00848-2)
Supplement: Supplementary file 1 — Supplementary material [file 41531_2024_848_MOESM1_ESM.docx]

Neurophysiological Gradient in the Parkinsonian Subthalamic Nucleus as a Marker for Motor Symptoms and Apathy

**Supplementary material**


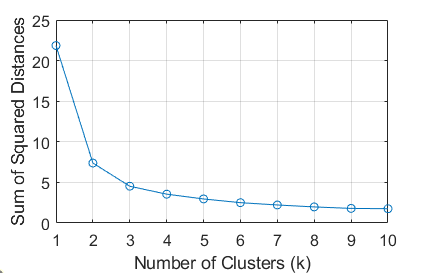


Supplementary figure 1: **Elbow method to detect the optimal number of clusters.** The graph illustrates the sum of squared distances when calculating k-means clustering for 1 to 10 number of clusters. The optimal number of clusters is selected visually where the sum of squared distances decrease slows down drastically. The “elbow” point was visually determined at k=3.

*
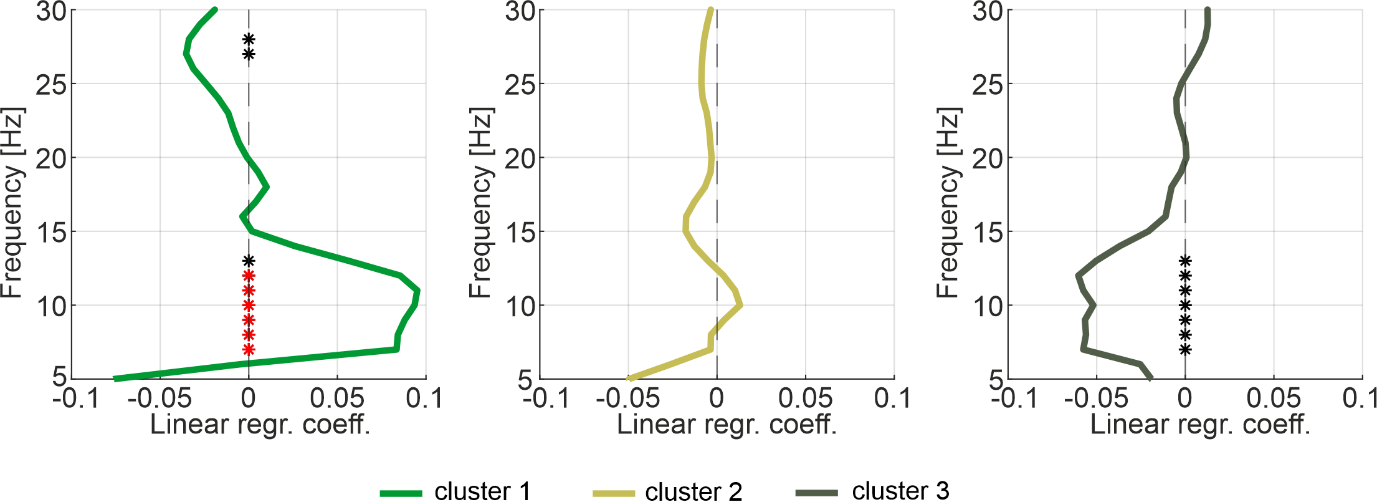
*

Supplementary figure 2. **Spectral gradient curves of main clusters.** Illustrates the power gradients along the mean trajectory of the three clusters (Figure 2) from 5 to 30 Hz. Power gradients were calculated based on the entire cohorts contact inside the STN pooled together. The spectral gradient values were compared to a surrogate gradient distribution derived by randomly shuffled power values of contacts. To control for multiple comparison, p-values were FDR corrected. The asterisks present the significant bins before (black) and after (red) FDR correction. For the mean axis of cluster 1, gradient values at the frequency bins between 7-13 Hz and 27-28 Hz are significant before FDR correction, whereas with FDR correction, the bins between 7-12 Hz are significant. For the mean axis of cluster 2, gradient values do not reach significance. For the mean axis of cluster 3, gradient values between 7-13 Hz are significant only before correction for multiple comparison. Note that non-standardized regression coefficients are shown. STN: subthalamic nucleus; FDR: false discovery rate


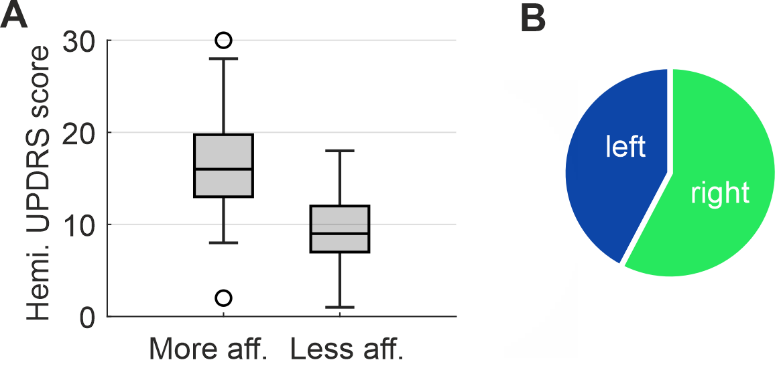


Supplementary figure 3: **Hemi-body MDS-UPDRS-III score distribution**. **A)** Illustrates the distribution of the hemi-body MDS-UPDRS-III scores (bradykinesia, rigidity, and tremor items) of the more and less affected side, if in subjects where both sides were assessed and one side had a higher score than the other. Subjects (n=59) had a median hemi-body MDS-UPDRS-III score of 16 ranging from 2 to 30 on the more affected side and of 9 ranging from 1 to 18 on the less affected side. **B)** Shows the ratio of hemispheres assigned as the more affected side on the left and right side (left: 25; right: 34 hemispheres). MDS-UPDRS: Movement Disorders Society Unified Parkinson’s Disease Rating Scale


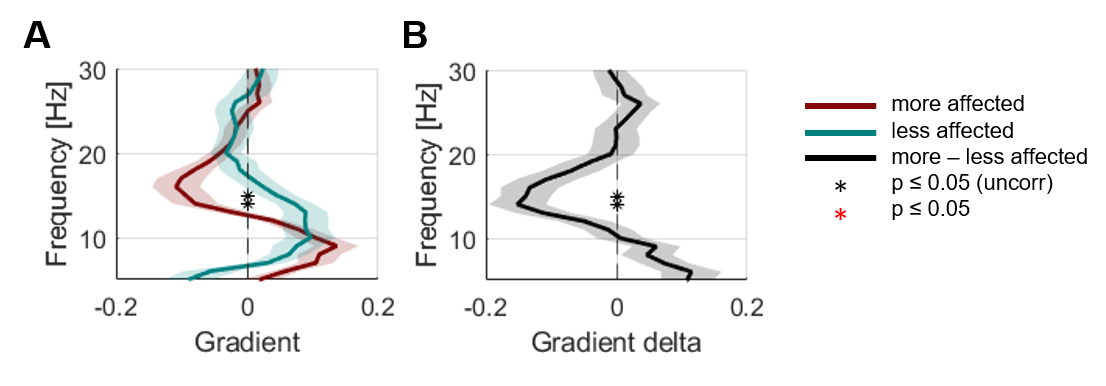


Supplementary figure 4: **Spectral gradients of more and less affected hemispheres.** **A)** Shows the mean ± SEM of the spectral gradients represented as linear regression coefficients from 5 to 30 Hz of the hemisphere contralateral to the more (dark red) and less (blue) affected hemi-body side only of subjects with spectral gradients in both sides. At the frequency bins 14 and 15 Hz, a significant different was seen without correction of p-values for multiple comparison. Note, a gradient toward right indicates increased power in the ventral STN, while a gradient toward left indicates increased power in the dorsal STN. **B)** Represents the difference in the gradient values between the more and less affected hemi-body side. While no significant cluster was present, significant p-values were seen for the single frequency bins at 14 and 15 Hz before correction for multiple comparison. SEM: standard error of the mean; STN: subthalamic nucleus.


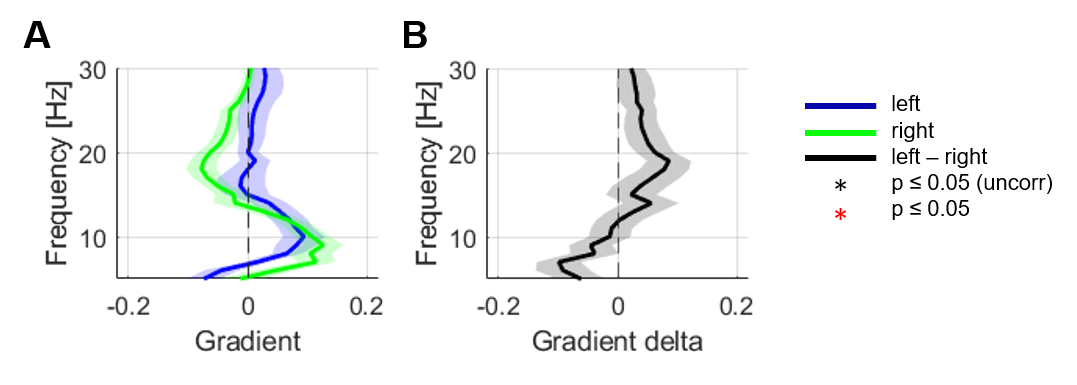


Supplementary figure 5: **Spectral gradients of the left and right hemispheres**. **A)** Shows the mean ± SEM of the spectral gradients represented as linear regression coefficients from 5 to 30 Hz of the left (blue) and right (green) hemispheres only of subjects with a left and right spectral gradient. Note, a gradient toward right indicates increased power in the ventral STN, while a gradient toward left indicates increased power in the dorsal STN. The gradients of the two sides do no differ significantly. **B)** Represents the difference in the gradient values between the left and right affected hemi-body side. The gradients of the two sides do no differ significantly. SEM: standard error of the mean; STN: subthalamic nucleus.


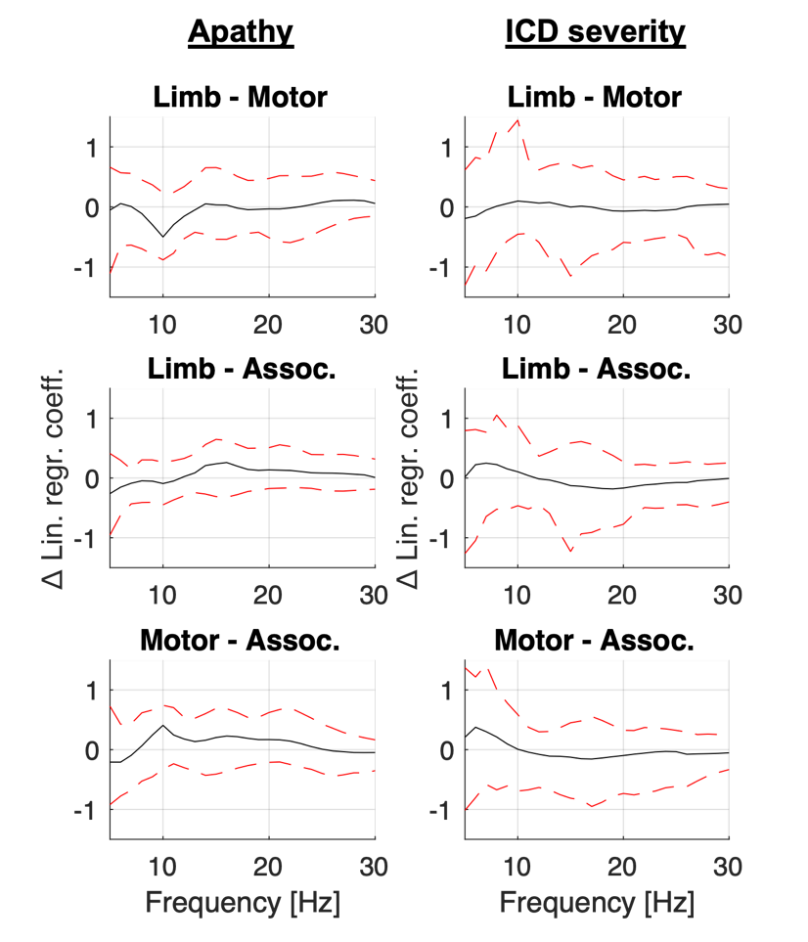


Supplementary figure 6: **Apathy score compared to power of functional subregions.** Shows the bootstrap result for the comparison of the in Figure 6 presented relationship between the apathy score and the power in the three functional subregions. Bootstrap samples were generated by resampling the spectral data and the scores with replacement. For each bootstrap sample, a linear regression fit was performed. The difference in the regression coefficients between the data to compare was computed for each bootstrap sample and the confidence intervals for the difference was estimated using the bootstrap distribution. The confidence interval for the difference was tested against zero. A significance level of 0.05 was used. Each figure presents the difference (black line) between the linear regression coefficients derived by two functional subregions and the 95-confidence interval (red dashed line) limits of the regression coefficient density distribution of 1000 bootstrap cycles, indicating no significant difference in the relationship of the apathy score with the power between regions.


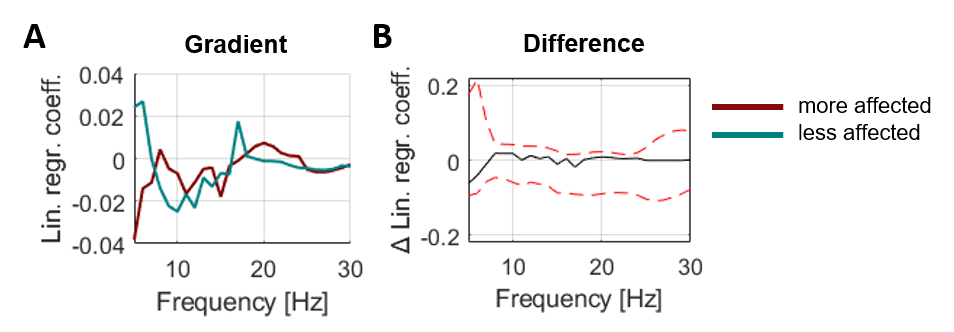


Supplementary figure 7: **Apathy score compared to spectral gradient of more and less affected hemisphere.** **A)** Illustrates the relationship between the Starkstein apathy score and the spectral gradient at each frequency bin between 5 and 30 Hz of the more affected hemispheres (dark red, n=13) and of the less affected hemispheres (blue, n=12). **B)** Shows the difference (black line) between red and dark curve presented in A) and the 95-confidence interval (red dashed line) limits of the regression coefficient density distribution of 1000 bootstrap cycles (bootstrap method, see Supplementary figure 6), indicating no significant difference in the relationship of the apathy score with the spectral gradient of the more and less affected hemisphere.


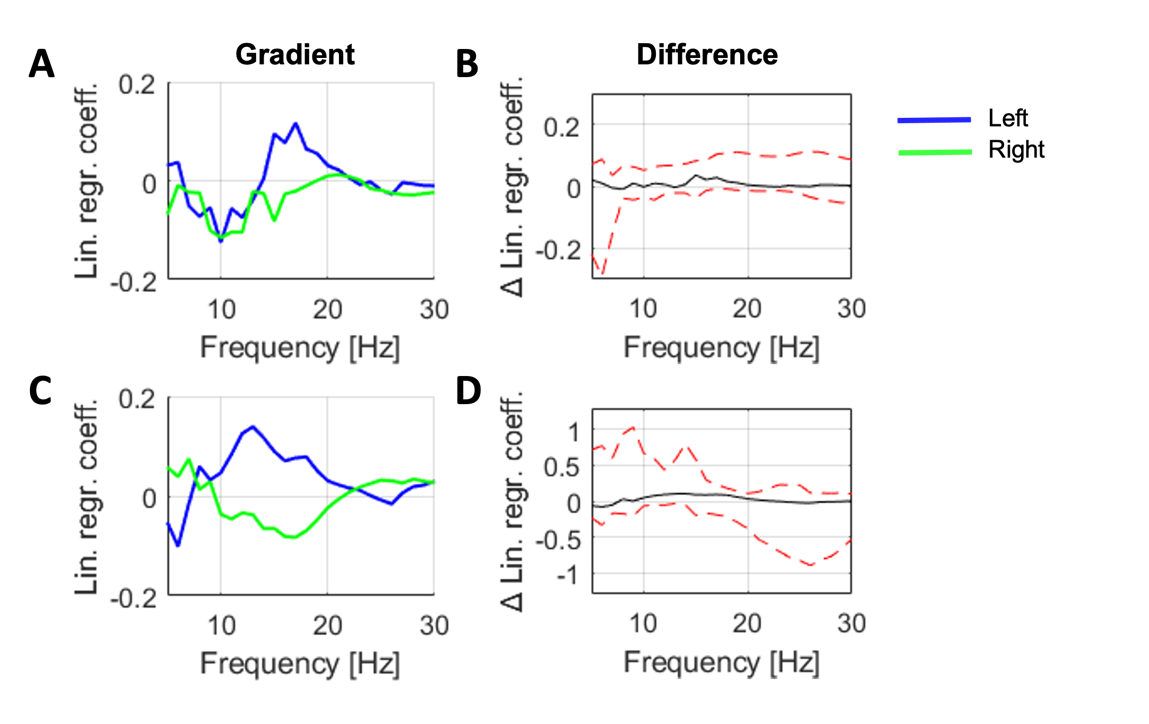


Supplementary figure 8: **Apathy score compared to spectral gradient of left and right hemisphere.** **A)** Illustrates the relationship between the Starkstein apathy score and the spectral gradient at each frequency bin between 5 and 30 Hz of the left hemispheres (blue, n=13) and of the right hemispheres (green, n=15). **B)** Shows the difference (black line) between blue and green curve presented in A) and the 95-confidence interval (red dashed line) limits of the regression coefficient density distribution of 1000 bootstrap cycles (bootstrap method, see Supplementary figure 6), indicating no significant difference in the relationship of the apathy score with the spectral gradient of the left and right hemisphere.


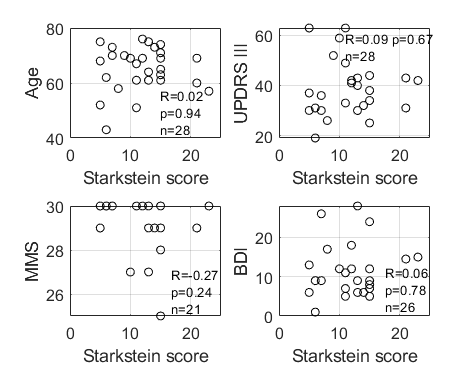


Supplementary figure 9: **Correlation of Starkstein score with other clinical and behavioral metrics.** The scatter plots illustrate the relationship and the Spearman correlation values between the Starkstein scores and the Age at the exam (rho=0.02, p-value=0.94), the total MDS-UPDRS-III score (rho=0.09, p-value=0.67), the Mini-Mental State (MMS) score (rho=-0.27, p-value=0.24) and the Beck Depression Inventory (BDI) score (rho=0.06, p-value=0.78). None of the correlations were significant.
